# Managing the stresses of group-living in the transition to village life

**DOI:** 10.1017/ehs.2022.39

**Published:** 2022-09-13

**Authors:** R. I. M. Dunbar

**Affiliations:** Department of Experimental Psychology, University of Oxford, Radcliffe Observatory Quarter, Oxford OX2 6GG, UK

**Keywords:** Homicide, hunter–gatherers, cultivators, bonding rituals, social institutions

## Abstract

Group living is stressful for all mammals, and these stresses limit the size of their social groups. Humans live in very large groups by mammal standards, so how have they solved this problem? I use homicide rates as an index of within-community stress for humans living in small-scale ethnographic societies, and show that the frequency of homicide increases linearly with living-group size in hunter–gatherers. This is not, however, the case for cultivators living in permanent settlements, where there appears to be a ‘glass ceiling’ below which homicide rates oscillate. This glass ceiling correlates with the adoption of social institutions that allow tensions to be managed. The results suggest (a) that the transition to a settled lifestyle in the Neolithic may have been more challenging than is usually assumed and (b) that the increases in settlement size that followed the first villages necessitated the introduction of a series of social institutions designed to manage within-community discord.

## Graphical abstract

**Figure:**
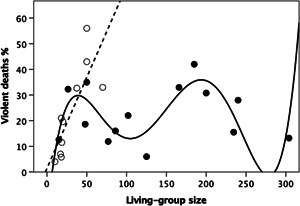

**Social media summary:** Group-living is stressful, with high levels of homicide. The transition to village life required social mechanisms to defuse these stresses

## Introduction

Living in large groups incurs significant costs. Across primates, for example, rates of within-group aggression increase with group size (Cowl & Shultz, 2017), as do behavioural indices of direct and indirect competition (e.g. day journey length and the time that has to be devoted to travel; Dunbar et al., 2009). In addition, the psycho-social stresses incurred by living in large groups have severe adverse consequences for female fertility that impact negatively on individual fitness and, as a direct result, limit social group size (Dunbar et al., 2018; Dunbar, 2018, 2019a; Dunbar & Shultz, 2021a). Except under extreme conditions on the edges of a taxon's distribution, these psycho-social stress effects appear to be a more serious limitation on group size than more conventional ecological constraints (Dunbar & Shultz, 2021a; see also Dunbar et al., 2009; Dunbar, 2019a).

If living in large groups is ecologically beneficial, then species must find ways to mitigate these costs, otherwise groups will be prone to fragmentation and oscillate around a lower equilibrium size (Dunbar et al., 2009, 2018; Dunbar, 2019a). In non-human primates, breaking through what amounts to a ‘glass ceiling’ set by these effects is achieved by the formation of coalitions that buffer group members against the pressures created by living in close spatial proximity to many other individuals (Dunbar, 2018, 2022a; Dunbar & Shultz, 2021b). The issue here is not about cooperation (which is more likely a consequence of living in stable groups) but about ensuring coordination among a group of individuals, where coordination refers to the maintenance of social cohesion such that the group continues to provide the intended fitness benefits for members (principally, protection against external threats) (Dunbar & Shultz, 2021b; Dunbar et al., 2018).

Humans are not exempt from these stresses. From time to time, San Bushmen feel the need to dispel (via a trance dance) what they call ‘star sickness’, a mysterious force that takes over a community, causing jealousy, anger, quarrels and a failure of gift-giving (Thomas, 2007). These pressures, they claim, pull people apart and damage community cohesion. Lee (1979) observed that disputes escalated in frequency when !Kung San gathered in large groups: serious disputes occurred once every two or three weeks at large waterholes that attracted 100–150 people, but only once every three or four months at smaller waterholes where only 30–50 individuals could comfortably camp together. In small-scale societies, a serious squabble between two people disturbs everyone. While studying the Siuai on Bougainville Island (Melanesia), for example, Oliver (1955) was informed that villages had to fission when they exceeded nine households, mainly because of quarrelling among the women. These effects continue to be evident in modern urban societies: increasing levels of stress owing to crowding and household density have adverse effects on health (Levy & Herzog, 1978; Lepore et al., 1991; Regoeczi, 2008; Riva et al., 2014) and are associated with increased rates of conflict and homicide (Pridemore, 2002; Nivette & Peres, 2021).

In the small scale, fission–fusion social systems characteristic of nomadic hunter–gatherers, these stresses are dissipated by dispersing the community (typically 100–200 individuals) into several small living groups (typically 30–50 in size: Zhou et al., 2005; Hamilton et al., 2007; Lobo et al., 2022), between which individual families are able to move when relationships with neighbours become too fractious (Wiessner, 2005). However, once a community switches into permanent settlement mode, it will face escalating stresses that, if unalleviated, will inexorably result in increasing costs (competition for resources, longer foraging journeys: Dunbar et al., 2009), greater difficulty in achieving consensus over management decisions (Dunbar & Sosis, 2018; Webber & Dunbar, 2020), and an increasing frequency of disputes with the risk of these spilling over into violence. Under these circumstances, families will leave to join smaller settlements, thereby setting an upper limit on equilibrium settlement size. Summarising a large amount of ethnographic and archaeological evidence, Alberti (2014) argued for a community size of 127 as the point at which these kinds of scalar stress start to become intolerable, with an upper limit at 158. If there is a benefit to living in settlements of larger size (e.g. protection against raiders: Johnson & Earle, 2000; Lienard, 2016), this will be possible only if solutions can be found that defuse these stresses and encourage good behaviour (Mathew & Boyd, 2011; Wiessner, 2019, 2020).

Early Neolithic populations in the Levant must have faced this problem as they moved from a huntering/gatherering lifestyle to one based on more permanent settlements associated with agriculture. Estimates of settlement size based on site area and number of dwellings suggest that the earliest settlements, associated with the brief Natufian culture (11,000–10,300 BP), were broadly within the range of modern hunter–gatherer living groups (~60 (Kuijt, 2000) or 75–100 (Belfer-Cohen & Goring-Morris, 2011)) and were associated with a mainly hunting-and-gathering economy. Once settlements were established, however, there was a rapid increase in community size to ~330 during the Pre-Pottery Neolithic phase A (10,300–9300 BP), rising to ~765 in the early Pre-Pottery Neolithic phase B (9300–8500 BP), and escalating to settlements of 3000–4000 over the following millennium (Kuijt, 2000). A similar sequence has been identified in the settlement patterns of Mesoamerica (Bandy, 2005). Although the archaeological record is necessarily silent on social behaviour, there are clear suggestions that when community sizes exceeded ~500 individuals, there was a rapid shift in structural organization associated with the emergence of elites (Hershkovitz & Gopher, 2008) and the appearance of specialised religious spaces and formal rituals (Carneiro, 1967; Adler & Wilshusen, 1990; Bandy, 2004; Dietrich et al., 2012).

Although conflict between females seems to be responsible for much of the stress-related infertility in mammals (Dunbar & Shultz, 2021a), the behaviour of young males can be critical in the kinds of large bonded groups that characterise humans and some primates. When males (and younger males, in particular) are deprived of social, economic and mating opportunities, they are prone to behaving in ways that both stress other group members (especially reproductive females) and threaten the stability and cohesion of the group. This is as true of the more social primates (chimpanzees, Wittig et al., 2008, 2016; baboons, Dunbar, 1984) as it is of humans (Boone, 1988; Raffield et al., 2017), and is often associated with high mortality rates. Under these circumstances, males are also likely to indulge in raiding neighbouring groups, which can result in poor inter-community relations as well as retaliation. Managing male behaviour may, thus, be crucial to maintaining an environment conducive to successful reproduction.

Disputes spilling over into violence and homicide have been an ever-present risk in both contemporary and historical small-scale human societies (Chagnon & Bugos, 1979; Lee, 1979; Knauft, 1987; Fausto, 2012; Walker & Bailey, 2013; Allen & Jones, 2016; Palmstierna et al., 2017). Archaeological evidence from pre-contact Australia indicates high levels of parry fractures (fractures of the forearm caused by raising the arm to parry blows: 10% of female skeletons and 8% of male skeletons), cranial depression fractures (from blows to the head by blunt instruments: 36% of women and 22% of men) and evidence of pre- and perimortem spearpoint wounds (Pardoe, 2016). Historical records indicate that internal conflict among the Viking communities in ninth to twelfth century Iceland was so severe that vendettas sometimes lasted a generation or more: in one such case (detailed in *Njáll's Saga*), all the adult males in a quarter of the extended families were killed (Dunbar et al., 1995). Revenge killing, sometimes involving innocent victims, was also widely reported among Australian Aboriginal groups in the late nineteenth and early twentieth centuries (Pardoe, 2016). In many of these cases, the killings were associated with accusations of witchcraft. A high frequency of witchcraft-motivated homicides (sometimes in pursuit of grudges) has been reported from New Guinea (Knauft, 1987). One likely explanation for this is that accusations of witchcraft reflect the difficulties that individuals have in coping with (and, especially, explaining) unexpected sickness and death (Dunbar, 2022b). That social stress and homicide may be intimately related is suggested by direct evidence from contemporary urban contexts, where homicide rates have been shown to correlate with the level of psycho-social stress experienced by the community (Vasquez et al., 2013; Pedersen et al., 2017).

On the strength of this evidence, I use data on overall homicide rates as a proxy for levels of social stress within a community to test three specific hypotheses that relate to the transition between a small-scale hunter–gatherer lifestyle and a larger scale settled village life: (a) in hunter–gatherer societies, homicide rates will increase as a function of living-group size, and in doing so set an upper limit to the number of people who can live together; (b) life in large permanent villages is only possible if the frequency of violent deaths can be kept below a critical threshold; and (c) this is achieved by introducing social institutions that allow conflict to be dampened by enhancing the sense of belonging to the community (community bonding).

To test the first two hypotheses, I use data on homicide rates from small-scale societies. Although a focus on within-community homicide might be preferable in order to be sure that we are dealing with internal social stresses, doing so greatly limits sample size. A broader definition that includes both within- and between-community homicide provides more statistical power, especially in respect of hypothesis (c). Nonetheless, I check the results against a smaller sample of within-group homicides. Since I am only interested in the first step from a hunter–gathering lifestyle to permanent settlements, I restrict my analysis to societies that typically have living groups <300 in size who pursue hunter–gatherer or slash-and-burn horticultural economies. I have not included pastoralist societies, partly because none are included in any of my source samples and partly because pastoralism is a late development that occurs after the transition to village settlements and tends to involve larger community sizes (Smith, 1992; Mace, 1993).

Note that I assume that homicide rates reflect demography rather than being a fixed characteristic of a culture. Let me emphasise that I am not here concerned with testing for all the many causes of homicide; rather, I use homicide rates as a proxy for social dyfunctionality, and hence I am only interested in whether or not homicide rates correlate with living-group size. Other factors may well be contributory, but for present purposes they constitute error variance. It is important also to be clear that we are not here concerned with why societies live in larger groups, or with whether this is associated with different kinds of economies, but simply with the *consequences* of living in groups of a particular size.

To test hypothesis (c), I examine 10 social institutions that, directly or indirectly, are likely to play a role in enhancing community cohesion – those that create a sense of belonging to the community. The focus is thus mainly on activities such as feasting, singing and dancing that are known to enhance social and community bonding (Tarr et al., 2015, 2016; Pearce et al., 2016, 2017; Dunbar, 2018, 2019b), mechanisms for managing the behaviour of the more volatile males (men's clubs, socially recognised leaders), and mechanisms for regulating marital arrangements (kinship, bridewealth, marital obligations, exogamy: Lévi-Strauss, 1969; Fox, 1983; Hughes, 1988). I have deliberately avoided institutions that are mechanisms of control (e.g. laws, punishments, policing, or mechanisms for resolving disputes such as duels), since these tend to be associated with much larger scale societies. I focus on social institutions that are widely recognised and hence likely to be reported in the ethnographic record if they are present. To make a convincing case, we need to show that these institutions appear in staged responses to changes in living-group size. If all institutions occur in all societies, or follow a highly correlated trajectory, this might be because they constitute a cultural assemblage that is unrelated to demographic or environmental conditions.

## Methods

I use data on homicide rates from three separate sources: (a) the percentage of adult deaths that were violent for 25 small scale societies, sourced from Keeley (1996), Bowles (2009) and Roser (2013), with all estimates checked against the cited original sources and corrected where they disagree; (b) the percentage of mortality attributable to within-community violence given by Gurven and Kaplan (2007) for a sample of seven societies that has only partial (~60%) overlap with sample (a); and (c) data on homicide rate per year per 100,000 population for 31 tribal societies given by Nivette (2011), which only partially overlap with the first two datasets. For the Nivette sample, I use only the data for small-scale ethnographic societies for which I can find an estimate of living-group size.

**Figure 1.**
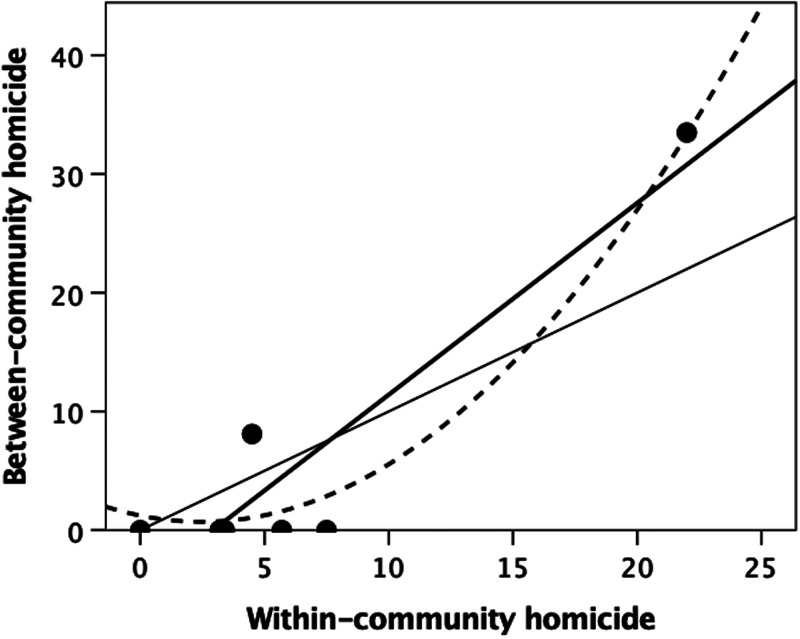
Percentage of all violent deaths that were due to between-community homicide (warfare) plotted against the percentage owing to within-community homicide (murder) for a sample of well-studied ethnographic societies. The thin solid line is the line of equality; the thick line is the best-fit linear regression (*r*^2^ = 0.939, *F*_1,5_ = 77.62, *p* = 0.0003); the dashed line is a quadratic fit (*r*^2^ = 0.972). Source: Gurven and Kaplan (2007).

Although few studies differentiate between- from within-community homicide, the evidence clearly indicates that the latter are in fact more common. Fry and Soderberg (2013) reviewed data on violent deaths in 21 ethnographic societies and concluded that, while some societies (such as the Australian Tiwi) did seem to engage in high levels of between-community violence (warfare), homicides were more likely to be the result of individual-level within-group violence. On average, 56% of all mortality in their sample was due to within-community violence, and only 38% to between-community violence. Knauft (1987), summarising nearly 400 homicides among the New Guinea Gebusi over a 40-year period in the first half of the twentieth century, noted that 54% of all homicides were perpetrated by a member of the victim's community, two-thirds of these being members of the same longhouse settlement. Similarly, Wiessner (2016), in reviewing 55 homicides from one !Kung community over a 40 year historical timespan, noted that ~95% of those involved were members of the same or neighbouring bands (i.e. members of the same community), with half of these being close family (including in-laws). Gurven and Kaplan (2007) were able to distinguish mortality owing to within-community violence from between-community mortality owing to warfare in six of the societies in their sample. The two variables are significantly correlated (Figure 1: *r*^2^ = 0.927, *F*_1,5_ = 77.62, *p* = 0.0003).

On the strength of these findings, I use the raw data on violent deaths as a reasonable proxy for within-community stress levels. The substantive statistical issue here is that broadening the definition of the dependent variable in this way is likely to introduce random error variance, and hence act conservatively by weighting the data against my hypothesis. If we obtain a statistically significant signal despite the error variance, it suggests that the effect is extremely robust.

The hypotheses I test concern the consequences of living together in the same space for sustained periods. I am therefore interested in the size of the living group (usually referred to as a band in the hunter–gatherer literature), not in higher level groupings such as temporary aggregations, clans or tribes that, in hunter–gatherers at least, are usually spatially dispersed and only come together for brief periods at irregular intervals for ritual or ceremonial purposes (e.g. corroborees in Australian Aboriginals). In order to ensure, in so far as possible, homogeneity of estimates, living-group size for hunter–gatherers was sourced in the first instance from Binford (2019); for societies not included in Binford's sample, estimates were sourced from Dunbar (1993) or primary ethnographic sources. For hunter–gatherers, the living group is taken to be the normal overnight camp group (band) and is equated with Binford's Group 1 category. Living groups for cultivators are equated with village size. Most of the latter practise swidden (or shifting) agriculture that can involve villages moving to a new site every few years as soils become exhausted; in between moves, however, villages are relatively static and community size stable (subject to the usual demographic effects of births and deaths). The data are given in Tables S1, S4 and S5.

Although all continents (other than Europe) are represented, the majority of the data derive from South America and New Guinea. In the percentage of violent deaths samples, there is an even distribution of hunter–gatherer and cultivator societies across continental regions (combining New Guinea with Australia: *χ*^2^ = 3.22, d.f. = 4, *p* = 0.521); neither living-group size nor the homicide rates vary significantly across continents for this sample (ANOVA – group size: F_5,16_ = 0.52, *p* = 0.755; homicide index: F_5,16_ = 0.98, *p* = 0.458). Of the 25 societies in the sample, only two pairs are culturally and linguistically related (Shuar and Achuar, both members of the Jivaro language group; and the Xilixana of northern Brazil and Yanomanö of southern Venezuela who are branches of the same cultural group). The first pair are classified in different subsets (one as hunter–gatherers, the other as village-based cultivators), while the second pair differ markedly in community size as well as violence rate. For sample 3 (the homicide rate data), a number of samples are for different Native American tribes from the Midwest, most of which have very similar economies and forms of social organisation. Although four (Blackfoot, Sioux, Cheyenne and Chippewa) of the six tribes represented belong to the same (Algonquian) language family, the other two (Kiowa/Comanche and Crow) belong to different language families, but behave very similarly. As a check, I analyse the data with and without this subsample. In sum, although the samples are small, it seems unlikely that the data are biased by region, economy or cultural phylogeny.

I use polynomial regression to determine the best fit regression equation for the data in each analysis. To determine the optimal solution, I plot the goodness-of-fit (*r*^2^) against the order of the polynomial and, following standard practice, seek the polynomial order where *r*^2^ asymptotes. As a check, I also ran a GAM (Generalized Additive Model) in R using the package *mgvc* (see the Supporting Information). The results were the same as those from the polynomial analyses.

To gain insight into the structural differences between these subgroups, I extracted from the primary ethnographic literature data on the presence/absence of 10 social institutions likely to play a role in managing violence and hence reducing social stresses. Definitions of the social institutions are given in Table S3; these data are also given in Table S1, with sources in Table S2.

In respect of managing conflict, I focus on mechanisms that allow conflicts to be managed before they get out of hand either by wise counsel or by male-bonding rituals (Dávid-Barrett & Dunbar, 2012, 2014; Glowacki & von Rueden, 2015; Wiessner, 2020). A great deal of attention has been devoted to dispute resolution in the recent literature (e.g. Wiessner, 2005, 2020; Glowacki & von Rueden, 2015; Garfield, 2021). Important as this might be, processes of dispute resolution are an after-the-fact mechanism for preventing disputes escalating into communal violence. By that stage, the damage in terms of social stress and its consequences has already been done. I am much more interested in processes designed to minimise the risk of disputes happening in the first place, and this is an issue about self-control, social bonding and facilitating the sense of obligation and commitment that arises from belonging to a community (Dunbar, 2012, 2021). This reflects the fact that, in face-to-face communities, adherence to the community mores is most effectively engineered by ‘bottom-up’ processes that foster a sense of obligation to others than by imposing punishment after an infringement has been committed (‘top-down’ processes, which can often have the opposite effect because being disciplined creates resentments) (Dunbar & Sosis, 2018; Dunbar, 2021, 2022b).

I include socially recognised leaders not in the sense of formal (typically inherited) tribal chiefs, but rather in the form of charismatic individuals whose advice, persuasion and influence are paid attention to because of their personal qualities of wisdom, eloquence, network connections, charisma and/or physical prowess (*sensu* Henrich & Gil-White, 2001; Glowacki & van Rueden, 2015; Garfield et al., 2020). In practice, this might include informal policing roles (although I did not include this as a criterion), but not arrangements for formal policing which imply the prior existence of laws and judges, both of which are largely absent in very small-scale societies. Note that the benefits of leadership (Garfield et al., 2020) are not considered because, even though there may be highly desirable fitness payoffs to the individuals concerned, these benefits are not relevant to maintaining social cohesion (and might even become a source of contention if their distribution becomes too unequal).

Since mating opportunities are a major source of frustration for younger males, especially in societies that practise polygamy (Boone, 1988; Fausto, 2012; Raffield et al., 2017), I include exogamy and alliances with neighbouring communities because, given the risk that small-sample bias statistical effects are likely to produce runs of male-biased offspring cohorts, exogamy (along with inter-community alliances) is one way of increasing the pool of available spouses without having to resort to kidnapping or raiding (see Chagnon, 1968; Fausto, 2012). Kinship beyond the immediate family and exogamy are also ways of creating a large cohort of interested parties who have a stake in the behaviour of the marital generation (Hughes, 1988) and who might therefore be willing to exert peer pressure on wayward individuals or protect the rights and security of victims of violence within marital relationships. This might be especially important in the context of exogamy where a spouse is socially isolated in the marital community and might be the butt of displaced aggression simply for that reason. Between-community bonding mechanisms may also be important for reducing the risk that internal conflict or frustrations spill over into external conflict through raiding expeditions. Being able to call on larger coalitions of allies can be crucial in this context – although this can, of course, cut both ways (among both Yanomamö and early medieval Icelandic Vikings, the men responsible for most within- and between-community murders are those who had the largest kin groups to back them up: Macfarlan et al., 2014; Palmstierna et al., 2017).

To determine whether these institutions correlate with group size, I first use *k*-means cluster analysis to partition the group size data into natural subsets representing different socio-demographic regimes. I then use Kendall's correlation to determine whether there is a monotonic increase in the frequency of these institutions across socio-demographic subgroups. Kendall specifically recommended this in cases where the abscissa is categorical in form but has a natural underlying continuum (Maxwell, 1961). I apply a one-tailed test because we are only interested in positive correlations. I use the results (1) to determine whether there is a general underlying increase in organisational complexity as group size increases and (2) to ascertain whether some institutions play a more important role at some stages than others.

Finally, I reanalyse Carneiro's (1967) data on organisational complexity in a large sample of ethnographic and historical societies to determine whether this pattern extends to a larger demographic scale. Carneiro (1968) lists some 60 social institutions that fall into two broad clusters: (a) craft specializations, structural organization above the family, formal leaders (chiefs), peace-keeping arrangements, specialist religious practitioners (e.g. shamans) and inter-community trade; and (b) administrative hierarchies, sumptuary laws, temples (i.e. organized religion with professional priests), markets, military conscription and state regulation of commerce.

## Results

**Figure 2.**
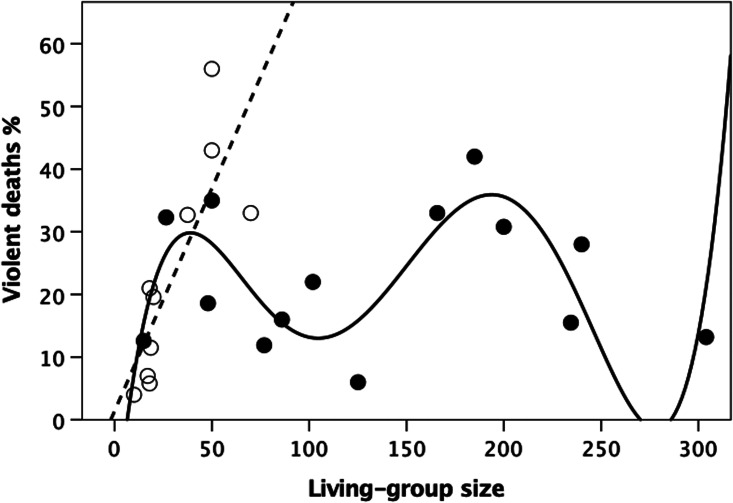
Percentage of mortality due to homicide (violence deaths) as a function of living group size, partitioned for hunter–gatherer tribes (unfilled symbols, dashed regression line) and village-based cultivators that depend largely on subsistence farming (filled symbols, solid regression line). Source: Table S1.

I first test for a statistical relationship between homicide rate and living-group size in each of the three mortality datasets. Figure 2 plots the percentage of deaths that involve homicide against living-group size in the ethnographic dataset, partitioning the data between hunter–gatherer and small scale village-living cultivator societies. Overall, there is an obvious bimodal pattern. Fitting polynomial regressions of different orders indicates that goodness-of-fit asymptotes at a fifth-order polynomial (Figure S1a). The best-fit least squares equation is:
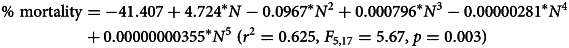
where *N* is the size of the living group. Double differentiating to identify inflexion points gives maxima at living-group sizes of 43 and 197, and minima at 112 and 282. Note that the upturn on the right is dependent on a single datapoint. Whether or not there is an upturn here is interesting, but not, in itself, germane to the claims being made. The main point is that there is a downturn from a living group of ~200, which would reach a minimum of 0% somewhere around a village size of 300.

Partitioning out the hunter–gatherers from village-based cultivators indicates that these two subgroups behave in very different ways. The hunter–gatherer data appear to form a simple linear relationship, whereas the cultivator societies form a sigmoid distribution similar to that found for the combined dataset. The best-fit regression equation for the hunter–gatherers, set through the origin, is:
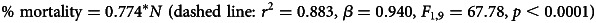
Living-group size does not generally exceed ~50 in hunter–gatherers (Zhou et al., 2005; Hamilton et al., 2007; Binford, 2019), and at this size violent deaths account for around half of all adult mortality. Extrapolating the regression equation implies that all deaths would be violent (in effect, no one would live long enough to reproduce) when living-group size was >134. In contrast, for the cultivators the proportion of violent deaths is again best fitted by a fifth-order polynomial (Figure S1b):
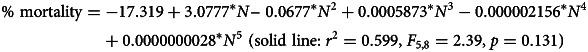
Aside from the intercept, the slope coefficients are of similar magnitude to those for the combined data. Double differentiating gives maxima at 39 and 194, with minima at 106 and 279.

Regression analysis can be problematic for data in proportion or percentage form owing to ceiling effects if the data cluster at 0 or 100% (a potential problem for the hunter–gatherers, but not for the village societies with their sinusoidal distribution). Although the meaning is less intuitive, one common solution is to use a log_10_ transformation of the odds ratio [log_10_(*p*/(1 − *p*))]: this circumvents the problem because this index varies between ±∞. Figure S2 plots this transform of the homicide data against living-group size. For the hunter–gatherers, the linear fit is significant (*r*^2^ = 0.629, *β* = 0.793, *F*_1,9_ = 15.24, *p* = 0.004) and very similar to that for the percentage data in Figure 2.

**Figure 3.**
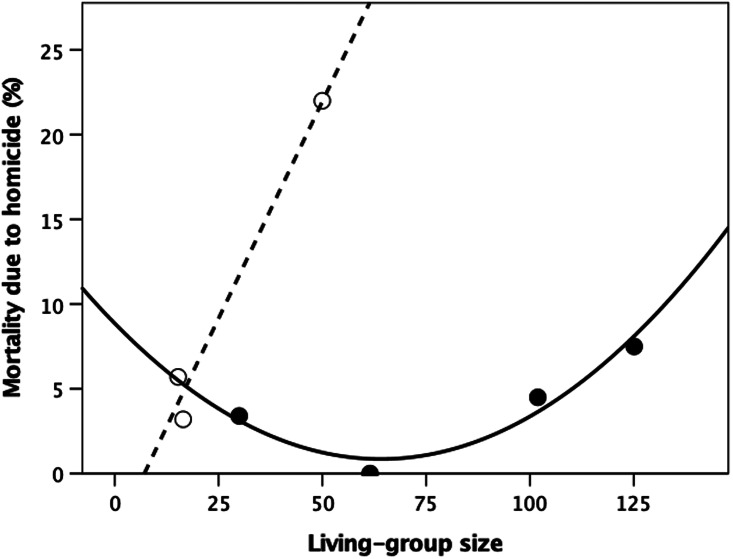
Mortality owing to within-community homicide plotted against living-group size for individual hunter–gatherer (unfilled symbols, dashed regression line) and village-living cultivators (filled symbols, solid line; quadratic fit). Source: Gurven and Kaplan (2007).

Gurven and Kaplan (2007) provide a dataset that offers a further test of these relationships because it differs from those in Figure 2 in two respects: (a) although the sample is smaller with a smaller range of living-group sizes, it only partially overlaps with that in Figure 2; and (b) it distinguishes between homicide owing to within-community and that owing to between-community conflict. Figure 3 plots the data for within-community homicide; the combined data for within- and between-community homicide are given in Figure S3. In both cases, the pattern is clearly very similar to that in Figure 2. For Figure 3, the best fit linear regression for the hunter–gatherer societies is:
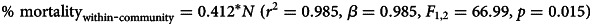
while that for the cultivators has a quadratic form, commensurate with the fact that the distribution of living-group sizes for this sample (30–125) covers only the middle section of the distribution in Figure 2:
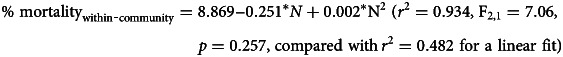
The parameter values are all but identical for the combined sources of homicide (Figure S3). A cubic equation would give a perfect fit:
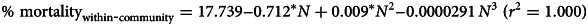
with parameter values that are very similar to the first four terms of the polynomial equation for Figure 2 (i.e. the terms that determine the left-hand side of the plot in Figure 2).

**Figure 4.**
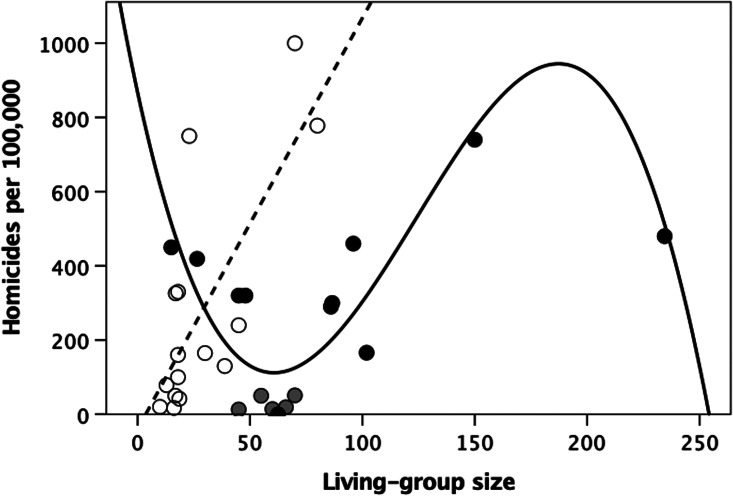
Homicide rate per year per 100,000 population for small-scale ethnographic societies plotted against mean living-group size. Unfilled circles and dashed linear regression line, hunter–gatherers; grey circles, North American plains Indians (other than the Blackfoot: the black symbol within this group) who alternate between small band hunter–gathering and large villages during the buffalo hunting season; filled circles, village-based cultivators. Source: Nivette (2011).

Figure 4 plots the data on homicides per 100,000 population per year for a sample of small-scale ethnographic societies collated by Nivette (2011). Once again, hunter–gatherer societies exhibit a linear relationship:
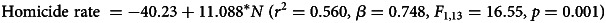
In contrast, the best-fit regression equation for the village-living cultivator societies is a third-order polynomial:
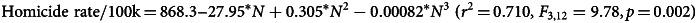
This regression includes five American Plains Indian datapoints (grey symbols) in addition to the Blackfoot, all of whom switch between small band hunter–gathering and large temporary villages (typically ~1000 people) during the annual buffalo hunt season. The plotted living-group size is that for their hunter–gatherer band, but these societies exhibit many adaptations for living in large groups (see Discussion). Excluding these societies does not change the result:
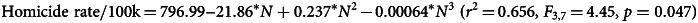
Note that the pitches of the two up-slopes in the polynomial regressions for the cultivator communities in Figures 2 and 4 are very similar. The two peaks in these regressions may be interpreted as breakpoints at which communities have to find ways to control escalating violence if community size is to increase further. Failing that, communities will oscillate around the breakpoint, unable to increase in size (as happens in Old World monkeys and apes: Dunbar et al., 2018).

To determine whether societies that live in larger living groups had social mechanisms designed to enhance community bonding so as to reduce the risk of violence, I first used *k*-means cluster analysis to partition the village-living cultivator societies into natural subsets based on living-group size. This partitioned these societies into three natural groups: those with living groups <50 where violence rates increase linearly, those with living groups of 50–150 where violence rates decline linearly, and those with living groups >150 where violence rates are again high. Although hunter–gatherer societies have living groups that are similar in size to the smallest cultivator category, I treat them as a fourth subset.

**Figure 5.**
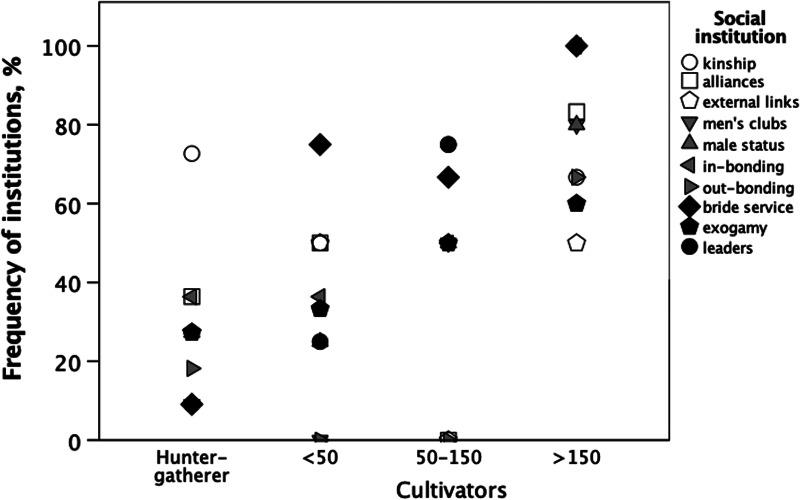
Percentage of tribal societies in the main ethnographic sample (sample 1) that exhibit different social institutions as a function of living-group size (with cultivator societies divided into three size categories plus hunter–gatherers). Unfilled symbols, traits with no correlation across community size; grey symbols, traits that increase exponentially across community size after an initial low threshold; filled symbols, traits with a linear increase across community size. Sample sizes are (left to eight): 11, 4, 4, 6 societies. For individual plots, see Figure S4. Source: Table S1.

Figure 5 plots the percentage of societies that possessed each of the 10 social institutions (see Tables S1 and S6; plots for individual institutions are given in Figure S4). Overall, there is a significant left-to-right increase in scores across the four socio-demographic groupings (Figure S5: mean Kendall's correlation for individual institutions, *τ* = 0.561; binomial test, *p*[_*τ* > 0 = 9/10 traits_] = 0.011; Fisher's meta-analysis confirms that the distribution of correlations is significantly more extreme than would be expected by chance if there was no underlying positive relationship: *χ*^2^ = 39.40, d.f. = 2 ×10 = 20, *p* = 0.006).

Although all of the correlations are positive, they in fact partition into three clear groupings on the basis of their patterns (Figures S4 and S5): (a) a set of institutions with Kendall's *τ* ≈ 0 that seem to characterise all societies, irrespective of size (kinship, alliances and friendly relations [between-group links] with neighbour communities); (b) a set that exhibit a linear increase with community size (charismatic leaders, marital obligations and exogamy); and (c) a set (male status competition, men's clubs, and within- and between-community bonding activities) that exhibit a J-shaped pattern (both hunter–gatherer societies and cultivators with living groups <50 exhibit low frequencies, with frequency increasing steeply when community size >50). This suggests a stepwise effect in which new mechanisms are progressively introduced to manage internal stresses as a way of breaking through successive glass ceilings.

**Figure 6.**
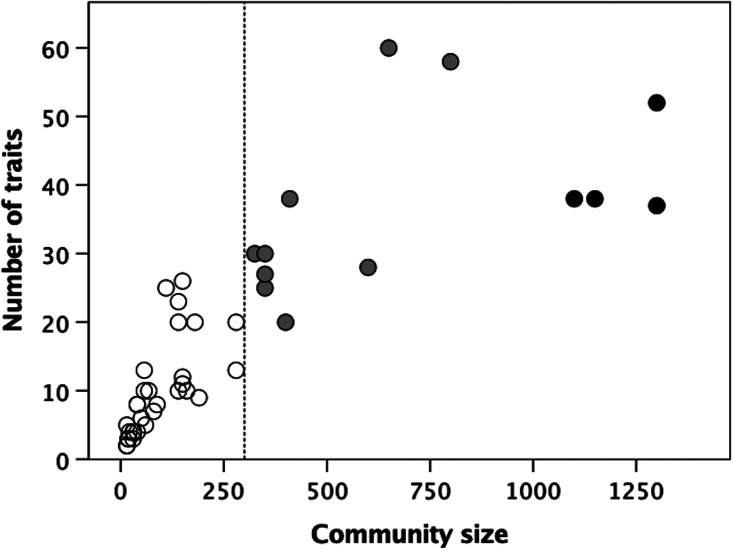
Number of social institutions as a function of mean community size for a sample of small scale societies. In contrast to the institutions shown in Figure 5, social institutions here include the presence of different craft and trade specialisations, laws and law enforcement mechanisms, formal religion and state regulation of trade. Bivariate *k-*means cluster analysis of the number of social institutions and community size yields an optimal division into three clusters (indicated by the different symbols). Dashed line demarcates a community size of 300. Data from Carneiro (1967).

To test the wider consistency of these results, I reanalysed Carneiro's (1967) data on 60 high-level social institutions in 44 ethnographic societies. As Carneiro observed, the number of institutions increases with the typical community size (Figure 6). However, a bivariate *k*-means cluster analysis suggests an optimum partition into *k* = 3 clusters (indicated by the different coloured symbols), with means at 10, 35 and 41 institutions centred at community sizes of 92, 471 and 1213. There seems to be a major phase transition at a community size of ~300 (indicted by the dotted vertical line) where the number of social institutions undergoes a step change from <20 to >20 traits. Those in the lower cluster only have structural and trade institutions (organisational institutions of the type listed under (a) in the final paragraph of the Methods), while those in the two higher clusters have institutions of the kinds listed in (b) (i.e. formal economic, administrative and judicial arrangements) in addition to those listed in (a). A second phase shift associated with a further step-increase in both the number of social institutions and community size appears to occur at a community size of ~1000 and seems to be associated with the appearance of formal religions (those with professional priests and temples, as opposed to shamans) and organisational structures associated with the rise of early small-scale polities (e.g. city states). In other words, these data seem to continue on a larger scale the same pattern observed for village-living cultivators.

## Discussion

Taken together, these data suggest that, to the extent to which homicide rates index within-community stresses, living in large groups is stressful and becomes increasingly so as group size increases. The hunter–gatherer data suggest that this sets a limit at around 50 or so on the number of individuals who can live together when there are no formal social mechanisms to defuse or manage conflict. At this limit, half of all adult mortality is due to homicide, effectively doubling the rate of mortality from conventional natural causes (i.e. disease, accident and old age). Given the high basal rates of mortality from conventional causes that are characteristic of ethnographic societies (Gurven et al., 2007; Hill & Hurtado, 2017), an increase in mortality rate of this magnitude would probably push the population below replacement rate and increase the risk of community extinction. Indeed, the rate at which these stresses increase appears to be sufficiently steep that all deaths would be the result of violence if the entire community of 100–200 individuals tried to live together as a single group for any length of time. One interesting implication of this is that it implies that the reason hunter–gatherers live in small dispersed subgroups has less to do with ecology and foraging than with the escalating costs of stress (just as seems to be the case in anthropoid primates; Dunbar & Shultz, 2021a).

If living in larger settlements is necessary to solve a key ecological problem such as raiding (Johnson & Earle, 2000; Abbink, 2009; Lienard, 2016) or to exploit highly specialised, monopolisable resources (e.g. salmon runs by Native Americans in the northwest USA: Smith & Codding, 2021), then this will only be possible if mechanisms can be found to mitigate the stresses involved. In the case of the American northwest, Smith and Codding's (2021) findings suggest that this is indeed so. Our understanding of the internal dynamics of human (and primate) social groups suggests that there are criticalities that set limits on the size at which social groups can function, given a set of social competences (Johnson, 1982; Dunbar, 2011, 2020; West et al., 2020; Webber & Dunbar, 2020). These seem to create a series of glass ceilings where further increases in settlement size are only possible if additional structural elements are introduced.

Broadly speaking, the activities involved (Figure 5) seem to be associated with formal rituals that enhance social bonding within and between neighbouring communities (who would usually be members of the same tribe). Charismatic, socially recognised leaders and men's associations/clubs seem to be especially important at larger group sizes (i.e. >100), perhaps because disputes between males can too easily escalate into violence when large numbers of allies can be recruited. Wiessner (2020) observed that bachelor clubs were introduced by the New Guinea Enga in an attempt to limit and channel the energy and volatility of the young adult males. Such clubs (which may take the form of age grades in some societies: Lienard, 2016) help to bond males (mutual obligation between close friends will make it less likely that disagreements get out of hand), while allowing disputes to be settled either through face-to-face discussion or constraints imposed by older, wiser counsel.

This switch from small informal groupings to larger structured ones parallels the finding that a formal management structure with a recognised leader invariably emerges in communities of practice in the business world once these exceed ~40 members in size (Webber & Dunbar, 2020). Of course, neither societies nor business organisations are obliged to increase their size: if there are no pressing reasons for having larger groups, they will prefer to maintain a size below the key threshold. Hutterites, for example, insist on splitting their communities so as to maintain their size below ~150 (community fissions over the last century averaged 168 individuals at fission: Alberti, 2014; Dunbar & Sosis, 2018). This is because they recognise that larger communities require organisational mechanisms such as laws, a judiciary and a police force to maintain order whereas communities of less than 150 can be run by strictly democratic means through face-to-face discussion, peer pressure and a sense of personal obligation.

The finding that structural features emerge at certain threshold community sizes (Figures 5 and 6) reinforces earlier suggestions to similar effect. Adler and Wilshusen (1990) found that ethnographic societies whose communities are smaller than ~250 people typically have generalised structures that are used for both domestic and ritual purposes, whereas those that live in larger communities invariably have separate, specialised ritual structures used only for formal meetings or ceremonies. This transition has also been documented in the archaeology of the Taraco Peninsula in Lake Titicaca, Bolivia. Bandy (2004) found that, around 1500 BC, mean village size (estimated from the number of dwellings) was about 127, with village fissions typically occurring at a size of around 170 (exactly the same size as in modern Hutterite community fissions: Dunbar & Sosis, 2018). By 1000 BC, mean village size had increased to about 275, with as many as a quarter of the villages containing more than 400 people. Shortly afterwards, a new religious complex, the Yaya–Mama tradition, made an appearance, associated with the rise of the Tiwanaku state. This cultural complex included a novel form of ceremonial public space (plastered sunken ball courts), decorated serving bowls, ceramic trumpets, incense burners and a distinctive style of stone sculpture (Bandy, 2004). This suggests that, at a size of around 400, more organised forms of religion become part of the social toolkit used to keep the lid on social fractiousness so as to allow larger communities to be stable enough to survive (Dunbar, 2022b). Carneiro (2000) has argued that, as the size of communities extends significantly beyond ~1000, social segmentation into moieties, clans or age-grades may be introduced to counter the divisive forces that otherwise threaten to destabilise very large communities. He pointed out that these often involve between-moiety relationships (such as formal arrangements for burying each other's dead) that help to bind the community together (cf. the between-community bonding rituals in Figure 5). Eventually, more formal political structures (legal systems, craft specialisations, formalised markets for the exchange of produce) arise in a process he likens to a phase transition.

The Plains Indians of the American Midwest provide clear evidence that the switch into more structured societies when living-group size exceeds that typical of hunter–gatherers is a phenotypic response to demographic conditions. Like many of these tribes, the Blackfoot alternated each year between small forager bands and very large (>1000) temporary village settlements for their annual spring buffalo hunt (Mandelbaum, 1979). While living in these large buffalo-hunt encampments, the individual band chiefs formed a council, with one of them elected as overall chief responsible for regulating the hunt and enforcing discipline, while the senior warriors from one or more bands were deputed to act as enforcers (i.e. a police force: Carneiro, 1967; Mandelbaum, 1979). If disputes did erupt, the parties concerned would be forced into a tipi to undergo a ‘sacred pipestem’ ritual that served to calm tempers. It may be this anticipated need for more formal management structures during the summer gathering that sets the Blackfoot apart from the other hunter–gatherers in the present sample in having formal leaders (chiefs) at band level (Table S1).

Large-scale alliances between living groups that create super-groups are often associated with trading arrangements designed to enable ecological risk management (Lehmann et al., 2014). However, they may have important additional social benefits. First, they create alliances that can be called upon when external threats escalate. That external threats have played a significant role, at least in recent human history, is indicated by considerable evidence for mass killings from Neolithic sites in the Near East (Hershkovitz & Gopher, 2008; Glencross & Boz, 2014; Knüsel et al., 2019), Europe (Frayer, 1997; Teschler-Nicola et al., 1999; Wahl & Trautmann, 2012; Meyer et al., 2015; Alt et al., 2020) and pre-contact North America (Walker, 1989; Milner et al., 1991; Willey, 2016). Massacres of Eskimo communities by NaDene Indians are recorded in both Eskimo oral tradition and the historical writings of contemporary European eyewitnesses, as well as the archaeological record (Melbye & Fairgrieve, 1994). Second, inter-community alliances offer an opportunity to expand access to a larger pool of spouses (insufficient numbers of whom is a principal source of social friction) and, at the same time, create a larger community of interested parties who might be willing to intervene when young males get out of hand. These possibilities have not been widely considered in the literature and would merit further exploration.

Human friendship and community bonding both rely on the same endorphin-based process (Depue & Morrone-Strupinsky, 2005; Dunbar, 2010; Machin & Dunbar, 2011; Loseth et al., 2014; Nummenmaa et al., 2016; Pearce et al., 2017, 2018) that underpins social bonding in primates more generally (Keverne et al., 1989; Martel et al., 1993). It is relevant that endorphin up-regulation and an enhanced sense of bonding are produced by many of the social activities that are commonly used in both within- and between-community rituals, including laughter (Manninen et al., 2017; Dunbar et al., 2021), singing (Pearce et al., 2015, 2016), dancing (Tarr et al., 2015, 2016), feasting (Dunbar, 2017; Dunbar et al., 2017; Nummenmaa et al., 2018), the rituals of religion (Charles et al., 2020, 2021) and storytelling (Dunbar et al., 2016). It is conspicuous that these forms of bonding activity appear to play an important role, especially in larger communities (Figure 5).

In summary, it seems that the stresses created by living in groups impose limits on the stability of groups once they exceed ~50 in size. These stresses can result in high levels of violence, although this is likely to be just the tip of a much larger iceberg that causes stress-related conditions that adversely affect female fertility (Dunbar et al., 2018; Dunbar, 2018; Dunbar & Shultz, 2021a) as well as the stability of social groups. Figure 2 suggests that there is a series of thresholds (or ‘glass ceilings’) that can only be breached if cultural mechanisms can be found to mitigate these stresses. Failing this, rates of violence will escalate to unsustainable levels, precipitating the break-up and dispersal of communities (Lee, 1979). The structural mechanisms for managing these stresses seem mainly to involve ways of enhancing within-group bonding (e.g. via community feasts and perhaps life-course ceremonies like puberty rituals and formal marriage arrangements) and imposing social (self-)discipline (especially on young males). The archaeological and ethnographic records would seem to add to this the suggestion that, when living groups exceed ~500 in size, organised religions emerge that are associated with formal rituals and specialised ritual sites (Bandy, 2004; Dunbar, 2022b). The analyses presented here suggest that there are very specific transition points in terms of community size at which these innovations occur. More detailed data on conflict rates, stress levels, network fragmentation and structural arrangements are needed to confirm this.

## Research transparency and reproducibility

All data on which the analyses are based are provided in the electronic Supplementary Information.

## Supplementary material

For supplementary material accompanying this paper visit https://doi.org/10.1017/ehs.2022.39. click here to view supplementary material
