# Supplementary material for: Managing the stresses of group-living in the transition to village life
Source: Evol Hum Sci. 2022 Sep 13;4:e40. doi: 10.1017/ehs.2022.39 (PMC10426039; doi:10.1017/ehs.2022.39)
Supplement: Supplementary file 1 [file S2513843X22000391sup001.docx]

**Managing the Stresses of Group-Living in the Transition to Village Life**

**R.I.M. Dunbar**

***Online Supplementary Material***

Table S1. Data for homicides sample. Sources are given in Table S3 below.

Society Region Violent Living-group Social institutions (1=yes; 0=no; blank: no information) ‡

deaths (%)* size †* 1 2 3 4 5 6 7 8 9 10

inbond outbond links alliance leader clubs status kinship bridal exogamy

**Hunter-gatherers**

Ache S. America 43.0 ¶ 50.0 0 0 0 0 0 0 0 0 0 0

Agta Philippines 7.0 ¶ 17.0 0 0 0 1 0 0 0 1 0 0

Anbara Australia 4.0 ¶ 10.0 0 1 1 0 0 0 0 1 0 0

Ayoreo S. America 19.6 20.0 # 0 0 0 0 0 0 1 1 0 1

Blackfoot N. America 33.0 70.0 1 1 1 1 1 1 1 1 0 0

Dobe !Kung Africa 18.6 18.6 1 0 1 1 0 0 0 0 0 0

Hadza Africa 3.2 16.5 1 0 0 0 0 0 0 1 0 0

Murgin Australia 21.0 ¶ 18.0 0 0 0 0 0 0 0 1 0 1

Shuar S. America 32.7 ¶ 37.5 1 0 0 1 0 0 1 1 1 1

Tiwi Australia 5.8 ¶ 18.0 0 0 0 0 0 0 0 1 0 0

Waorani S. America 56.0 ¶ 50.0 0 0 0 0 0 0 0 0 0 0

**Village-based cultivators**

Angorr New Guinea 11.9 ¶ 76.9 1 0 0 1 0 0 0 0 0

Dugum Dani New Guinea 15.5 ¶ 234.5 1 1 1 1 1 1 1 0 1 1

Gebusi New Guinea 32.3 ¶ 26.5 0 0 1 0 0 0 0 1 0 0

Huli New Guinea 13.2 304.0 1 1 1 1 1 1 1 1

Mae Enga New Guinea 18.6 ¶ 48.0 0 0 1 1 1 0 0 0 1 1

Modoc N. America 12.6 ¶ 15.0 0 0 1 0 0 0 0 1

Kayapo S. America 30.8 ¶ 200.0 1 1 0 1 1 1 1 1 1 0

Achuar S. America 42.0 ¶ 185.0 1 0 0 1 1 1 1 0 1 1

Hiwi S. America 33.0 ¶ 165.8 1 0 0 1 1 0 0 1 0

Tsimane S. America 6.0 ¶ 125.1 0 0 0 1 0 0 1 0 0

Wari S. America 28.0 240.0 1 1 1 0 1 1 1

Xilixana S. America 16.0 86.0 0 0 0 1 1 1 1 1 1 1

Yanomamö S. America 22.0 ¶ 101.9 1 0 0 1 1 1 1 1 1 1

Arawete S. America 35.0 50.0 1 0 0 0 0 0 1 1 1 0

* Sources are given in Table S2.

¶ Estimate is for adult mortality only. Ayoreo, Wari and Xilixana values are based on data from all ages; Blackfoot and Dobe !Kung are unspecified.

# Based on an estimated population in mid-1940s of ~500 divided between 25 bands (given by Bugos 1985). Omitting this case does not change the results.

† **Living-group size** refers to the average number of individuals of all ages and both sexes living together in the same space overnight. It corresponds to the society’s typical living arrangements. For hunter-gatherers, this refers to overnight camp groups (sometimes referred to as bands); it does not refer to or include hunting camps (which are usually male-only) or the periodic gatherings associated with communal rituals such as puberty or marriages. For horticultural societies, it refers to the size of settlements or villages; these are usually stable for longer than hunter-gatherer camps, though they may be moved from time to time as fields are exhausted or raiding becomes intolerable. Both usually exhibit a significant degree of membership stability (allowing for births and deaths), at least on the scale of months. Where I sourced data from Binford (2019), I use his GROUP1 variable. Where only a range is given, I use the median value in all analyses.

‡ The social institutions are defined in Table S3. 1: within-group bonding rituals; 2: between-group bonding rituals (e.g. feasts); 3: friendly between-group relationships; 4: formal between-group alliances; 5: charismatic leaders; 6: men’s clubs; 7: male status rivalry; 8: extended kinship; 9: brideprice/service; 10: exogamy. Sources: see Table S3 (final column).

Table S2. Sources for data in Table S1.

Society Ecology % violent deaths Living-group Structural traits (Table S1)

size

Ache [pre-contact] hunter-gatherer Hill & Hurtado 1996 Binford 2019 Hill & Hurtado 1996

Agta [1936-1950] hunter-gatherer Headland 1989 Headland 1987 Minter 2017

Anbara [1940-1950] hunter-gatherer Bowles 2009 Binford 2019 Meehan 1975

Ayoreo [1920-1979] hunter-gatherer Bugos 1985 Bugos 1985 Bugos 1985, Diez & Salzano 1978

Blackfoot [1858] hunter-gatherer Roser 2013 Binford 2019 Mandelbaum 1979

Dobe !Kung hunter-gatherer Bowles 2009 Dunbar 1993 Lee 1972, 1979

Hadza hunter-gatherer Gurven & Kaplan 2007 Binford 2019 Marlowe 2002, 2010

Shuar hunter-gatherer Roser 2013 Lingarde et al. 2004 Mader & Gippelhauser 2000

Murngin [1910-1930] hunter-gatherer Bowles 2009 Binford 2019 Hiatt 1965

Tiwi [1893-1903] hunter-gatherer Roser 2013 Binford 2019 Hart 1930

Waorani [pre-contact] hunter-gatherer Walker & Bailey 2013 Beckerman et al. 2009 Rival 1993; Macfarlan et al. 2018

Achuar cultivator Walker & Bailey 2013 Orr et al. 2001 Espinoza 2020; Mader & Gippelhauser

2000

Anggor cultivator Huber 1973 Huber 1973,1980 Huber 1973, 1980

Dugum Dani cultivator Heider 1970 Somerville et al. 2017 Heider 2017, Shankman 1991

Gebusi [1940-1982] cultivator Knauft 1987 Dunbar 1993 Knauft 1987

Hiwi [pre-contact] cultivator Walker & Bailey 2013 Gurven et al. 2000 Arcand 1973; Gurven et al. 2000;

Hill et al. 2007

Huli cultivator Keeley 1996 Glasse 1968 Glasse 1968

Kayapo [1935-1953] cultivator Werner 1980 Gross et al. 1979 Werner 1980; Turner 2003

Werner 1980

Mae Enga cultivator Keeley 1996 Dunbar 1993 Meggitt 1957

Modoc [‘pre-modern’] cultivator Bowles 2009 Binford 2019 Sobel & Bettles 2000

Tsimane [1950-1989] cultivator Walker & Bailey 2013 Binford 2019 von Rueden 2011; von Rueden et al.

2014

Wari [pre-contact] cultivator Walker & Bailey 2013 Conklin 1995 Tung 2007;

Xilixana [pre-contact] cultivator Walker & Bailey 2013 Walker & Hill 2014 Early & Peters 2000

Yanomamo cultivator Walker & Bailey 2013 Dunbar 1993 Chagnon 1968; Macfarlan et al. 2014

Arawete [pre-contact] cultivator/HG Walker & Bailey 2013 De Castro 1992 De Castro 1992

Table S3. Criteria for the 10 social institutions listed in Table S1.

Trait Definition

1. within-group bonding rituals within-community feasts, communal singing, ritual dances (e.g. trance dance); helps to bind the group together

and reduces stress levels through the release of endorphins

1. between-group bonding rituals formal feasts, communal singing and dancing involving neighbour living-groups; provides a pool of potential

spouses, as well as a set of interested parties willing to exercise some control over the disruptive behaviour of

young married males

1. between-group links friendly or cooperative relationships with at least one neighbouring living-group, annual meetings (e.g.

corroborees); provides a pool of potential spouses, as well as a set of interested parties willing to exercise some control over the disruptive behaviour of young males

1. between-group coalitions coordinated arrangements (alliances) for raiding or defence with specific neighbour living-groups; provides a

pool of potential spouses, as well as a set of interested parties willing to exercise some control over the

disruptive behaviour of young males

1. charismatic leaders one or more individuals informally recognised or elected as a non-hereditary living-group leader (e.g. village

headman, charismatic leader, ‘Big Man’ or shaman where status depends on the knowledge, wisdom, power

or network size of the individual); provides an authority figure able to exert some control over the disruptive

behaviour of younger males, either by imposing discipline or by providing wise counsel

1. men’s clubs men’s secret associations/talking-shops, ritual spaces or houses for men-only use; contexts in which conflicts

between males can be defused and/or males bonded with each other in ways that enhance a sense of obligation

1. male status men explicitly compete with each other for status, or perform some act (e.g. killing an enemy) for recognition;

indicative of implicit authority to impose discipline or guidance on younger males

1. extended kinship kinship as an explicit organising principle for within community relationships (e.g. marriage rules), dependent

on the capacity to name specific degrees of relatedness for specific individuls; creates a network of people

with vested interests in managing conflict within the community

1. marital services men required to pay brideprice to bride’s family on marriage, or live and work for bride’s family for period

after marriage; helps build a network of interested parties willing to exercise control over the disruptive

behaviour of young males

1. exogamy marriages preferably or mainly between individuals from different living-groups/communities; helps build a

network of interested parties willing to exercise control over the disruptive behaviour of young males

Table S4. Data on for percentage of all mortality due to homicide, including both within-community and between-community (i.e. warfare) ,mortality given by Gurven & Kaplan (2007) for individuals >15 years of age.

Society Ecomomy Living-group % of homicide due to: Source for

size within-community all sources group size

conflict of conflict

Hadza hunter-gatherer 16.5 3.2 3.2 Binford 2019

!Kung hunter-gatherer 18.6 11.7 Binford 2019

Agta hunter-gatherer 17.0 3.0 Binford 2019

Australian aboriginals (mean) hunter-gatherer 15.3 5.7 5.7 Binford 2019

Ache (forest) hunter-gatherer 50.0 55.5 22.0 Binford 2019

Hiwi cultivator 165.8 30.2 Gurven et al. 2000

Tsimane cultivator 125.1 7.5 7.5 Binford 2019

Machiguenga cultivator 30.0 3.4 3.4 Johnson & Behrens 1982

Bakairi cultivator 61.5 0.0 0.0 Picchi 1995

Yanomamo cultivator 101.9 12.6 4.5 Dunbar 1993

Table S5. Data and sources for analysis of homicide rates

Society Region Economy Homicide rate Living-group Living-group

/year/100,000 * sources

------------------------------------------------------------------------------------------------------------------------------------------

**Hunter-gatherers**

Eskimo Greenland hunter-gatherer 17 16.2 Binford 2019

Inuit N. America hunter-gatherer 100 18.0 Binford 2019

Piegan Blackfoot N. America hunter-gatherer 1000 70.0 Binford 2019

Yoruk N. America hunter-gatherer 240 45.0 Binford 2019

Paiute N. America hunter-gatherer 130 38.8 Binford 2019

Kiowa-Comanche N. America hunter-gatherer 14 60.0 Binford 2019

Cheyenne N. America hunter-gatherer 13 45.0 Binford 2019

Sioux (Assiniboine) N. America hunter-gatherer 50 55.0 Binford 2019

Crow N. America hunter-gatherer 19 66.0 Binford 2019

Blackfoot (Montana) N. America hunter-gatherer 51 70.0 Binford 2019

Chippewa N. America hunter-gatherer 750 23.0 Binford 2019

Apache N. America hunter-gatherer 165 30.0 Binford 2019

Shoshone N. America hunter-gatherer 79 12.9 Binford 2019

Hewa New Guinea hunter-gatherer† 778 80.0 Steadman 1971

Andamanese S. Asia hunter-gatherer 20 10.0 Binford 2019

Agta S. Asia hunter-gatherer 326 17.0 Headland 1987

Murngin Australia hunter-gatherer 330 18.0 Binford 2019

Tiwi Australia hunter-gatherer 160 18.0 Binford 2019

Baka Africa hunter-gatherer 50 16.8 Binford 2019

!Kung San Africa hunter-gatherer 42 18.6 Binford 2019

**Village-based cultivators**

Modoc N. America cultivator 450 15.0 Sobel & Bettles 2000

Xilixana S. America cultivator 290 86.0 Walker & Hill 2014

Yanomano S. America cultivator 166 101.9 Dunbar 1993

Telefolmin New Guinea cultivator 740 150.0 Brumbaugh 1980

Dugum Dani New Guinea cultivator 480 234.5 Somerville et al. 2017

Manga New Guinea cultivator 460 96.0 Cook 1967

Gebusi New Guinea cultivator 419 26.5 Knauft 1987

Tauade New Guinea cultivator 320 45.0 Hallpike 1986

Mae Enga New Guinea cultivator 320 48.0 Meggitt 1957

Eipo New Guinea cultivator 300 88.6‡ Schiefenhovel 1998

Semai S. Asia cultivator 0 62.5 Robarchek & Robarchek 1992

---------------------------------------------------------------------------------------------------------------------------------------

* All homicide data are from Nivette (2011).

† In contrast to other New Guinea tribes, the Hewa live mainly by hunting wild pigs and other animals and gathering (of wild ambrea fruit and pandanus, and cultivate only yams; they also live is semi-isolated houses that form dispersed communities (Steadman 1971).

‡ geometric mean of minimum (30) and maximum (250) values.

**Goodness-of-fit for polynomial regression of different order for Fig. 2.**


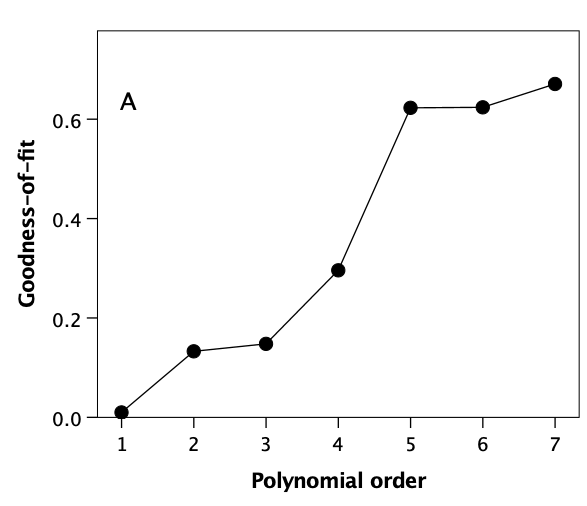

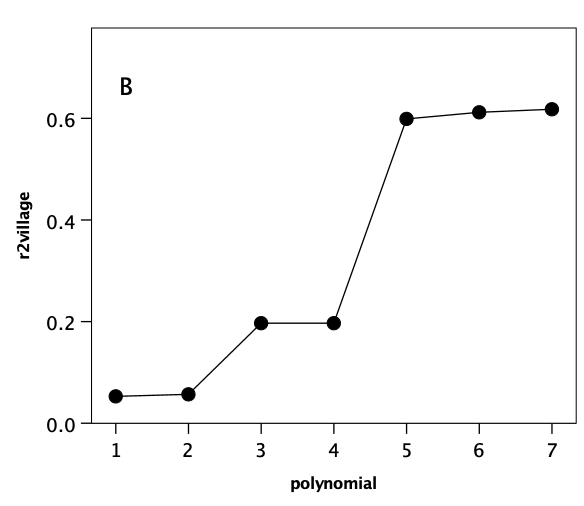


**Figure S1**

Goodness-of-fit (indexed as r^2^) for polynomial regressions of different order for percentage of violent deaths plotted against living-group size for (a) the combined dataset and (b) cultivator societies only. The optimal equation is identified by the order at which the goodness-of-fit asymptotes, since there is little further improvement in fit after this point. In both cases, the optimal regression is a fifth order polynomial.

**GAM analyses of the polynomial data**

As a check on the polynomial regressions in Fig. 2, I ran a GAM (Generalized Additive Model) analysis on the same data using the R package *mgcv*. The results yielded distributions of similar shape that did not differ from those obtained for the polynomial analyses. The goodness-of-fit for the combined data and the horticultural societies only were:

1. Combined data: r^2^_adj_ = 0.880, N=22, F_8.60,8.95_ = 9.741, p = 0.0704
2. Horticulturalists only: r^2^_adj_ = 0.502, N=12, F_4.66,5.44_ = 4.029, p = 0.0124

The goodness-of-fit is slightly improved, but in neither case are the results a significant improvement on those obtained by a polynomial regression.

**Does running a regression on percentages introduce a confound?**

**
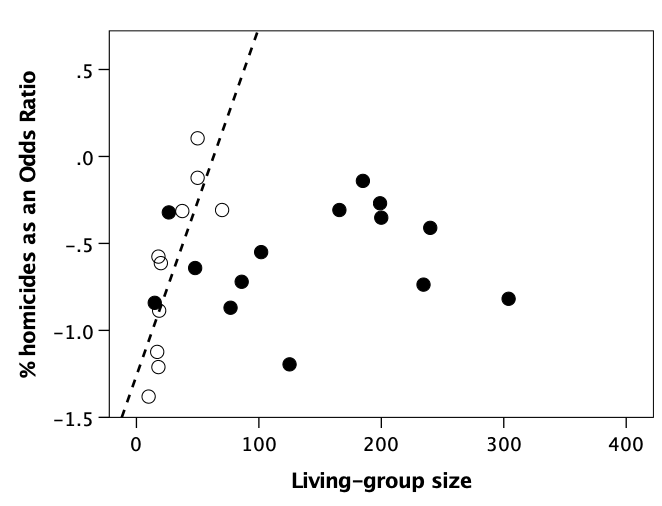
**

**Figure S2**

To avoid a ceiling effect through using percentage data in a linear regression, the percentage of deaths due to violence can be transformed to an Odds Ratio [log_10_(p/(1-p))]: this circumvents the problem because the data then vary between ±∞. The best-fit regression for the hunter-gatherer data (unfilled symbols) is a linear regression that is very similar to that for Fig. 1 (r^2^=0.629, β=0.793, F_1,9_=15.24, p=0.004). The problem does not arise with village-based horticulturalists, whose data do not approach the ceilings at 0% and 100%.

**Gurven & Kaplan (2007) data for mortality due to all forms of violence (i.e. within-community and between-community deaths combined).**

**
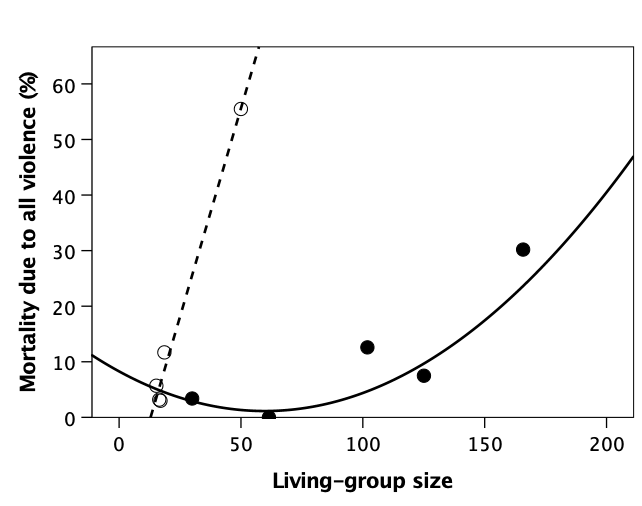
**

**Figure S3**

Mortality due to all sources of homicide (including that due to between-community warfare as well as within-community conflict) plotted against living-group size for individual hunter-gatherer (unfilled symbols, dashed regression line) and village-living horticulturalists (filled symbols, solid line). Source: Gurven & Kaplan (2007)

In both cases, the pattern is very similar to that for homicide due only to within-community conflict (Fig. 3) and to the larger (and only partially overlapping) sample in Fig. 2. The best-fit regression for hunter-gatherer societies is:

Homicide_All_ % = 0.878*N

(r^2^=0.923, standardised β=0.923; F_1,3_=4.79, p=0.009), where N is living-group size. For village-based cultivators, a quadratic equation provides the best-fit because the data cover only the middle range of the data in Fig. 2:

Homicide % = 8.29 – 0.239*N + 0.002*N^2^

(r^2^=0.881, F_2,2_=7.38, p=0.119).

Table S6. Percentage of societies in each demographic category that exhibit each of the 10 social traits for Fig. 3

Trait* inbond outbond links alliance leader clubs status kinship marital exogamy

Hunter-gatherers 36 18 27 36 9 9 27 73 9 27

Horticulturalists:

<50 36 0 50 50 25 0 25 50 75 33

50-150 50 0 0 0 75 50 50 75 67 50

>150 100 67 67 50 100 80 80 67 100 60

Kendall’s τ 0.913 0.183 0.183 0.333 1.000 0.667 0.667 0.000 0.667 1.000

p (1-tailed) 0.035 0.359 0.359 0.248 0.087 0.087 0.087 0.500 0.087 0.087

* Traits as defined in Table S3. The source data are in Table S1.

**Figure S4**

Individual plots for each social trait as a function of socio-demographic category

**
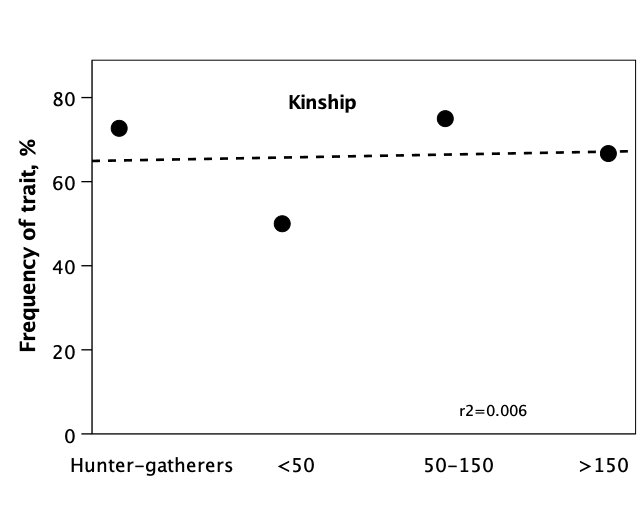

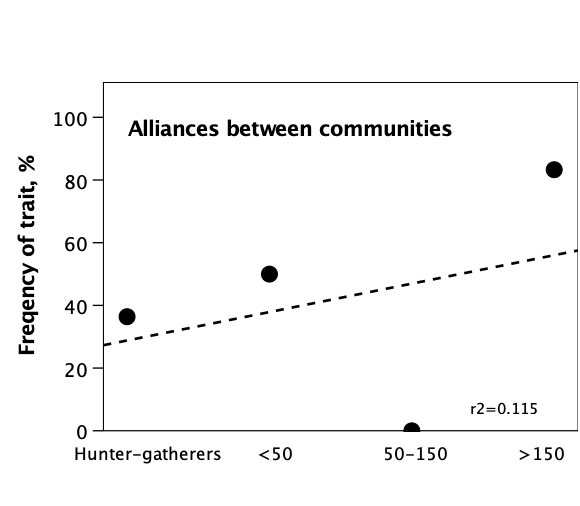

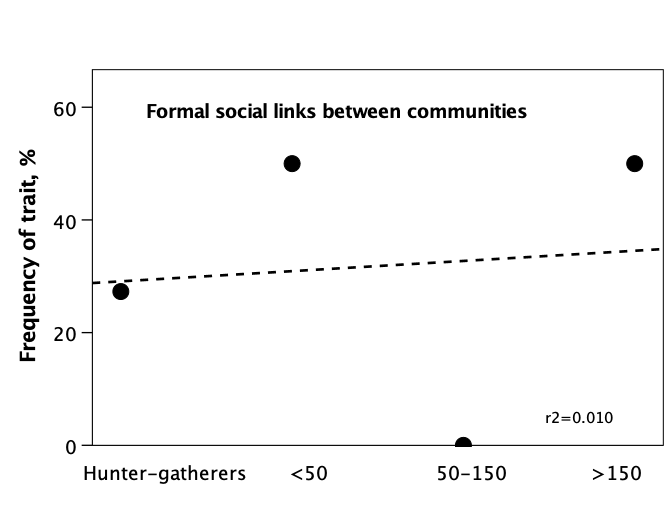
**

**
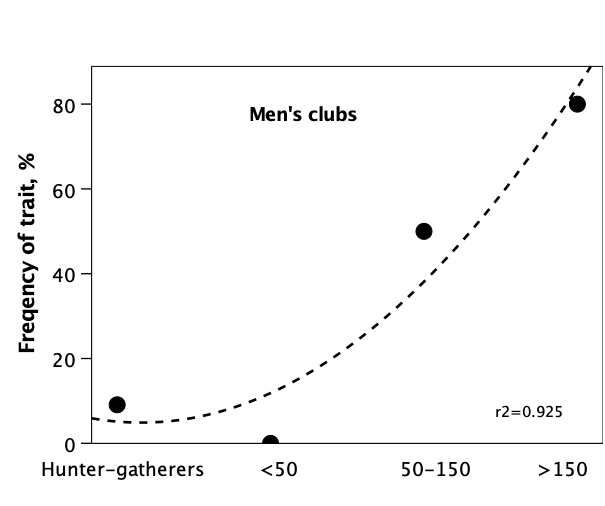

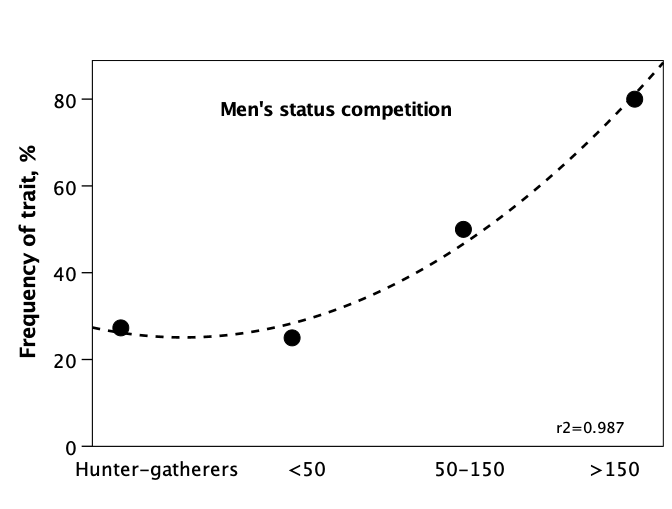
**

**
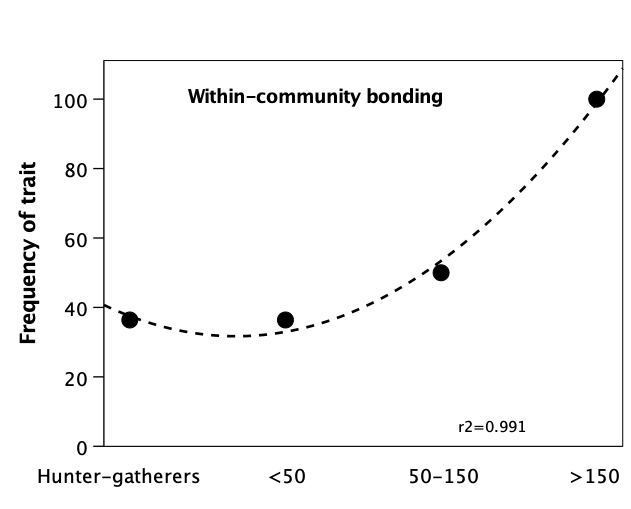

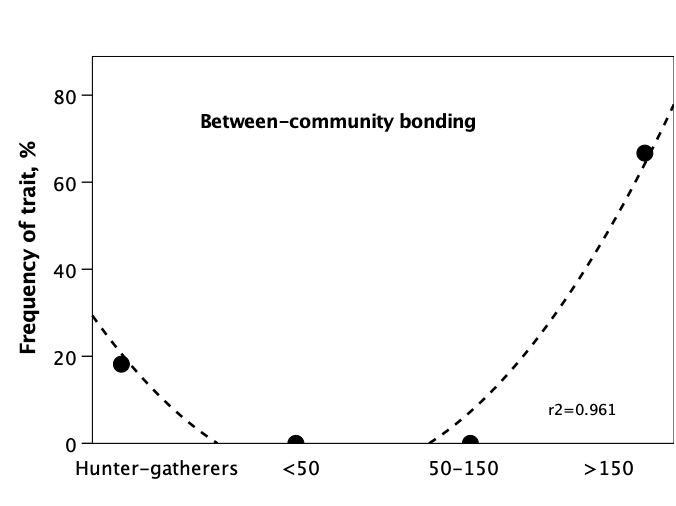
**

**
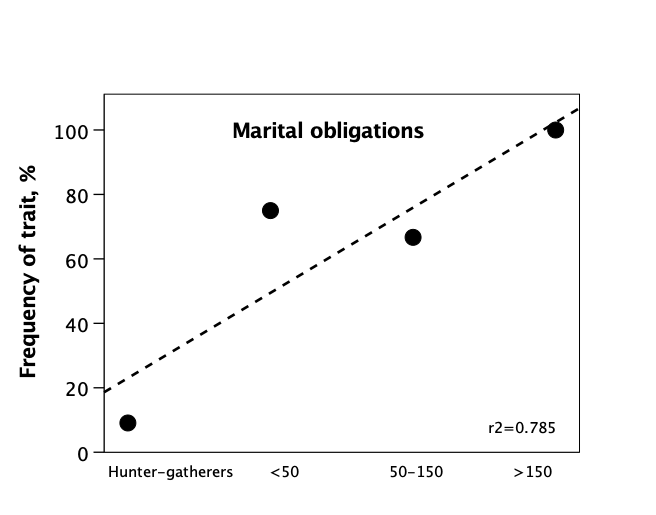

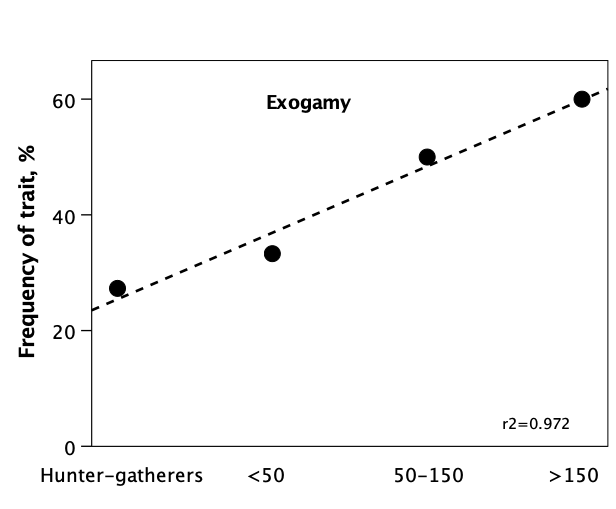

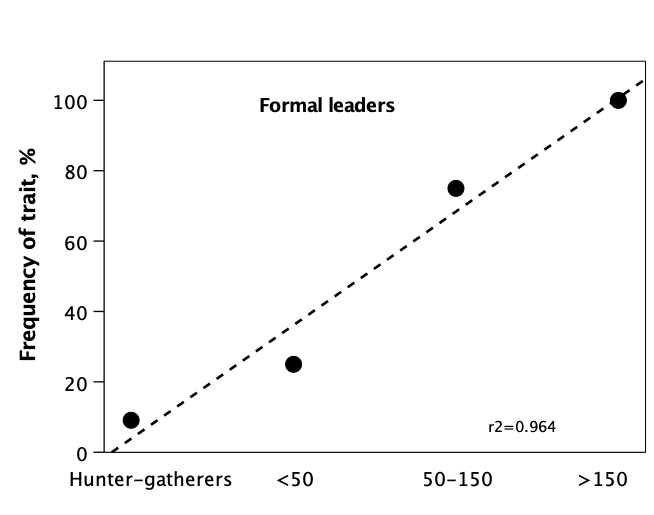
**

**Figure S5**

Distribution of Kendall’s τ correlations between percentage of societies exhibiting a particular social institution across the four socio-demographic categories for the social institutions shown in Fig. 5. N=4 sociodemographic categories in each case.

**
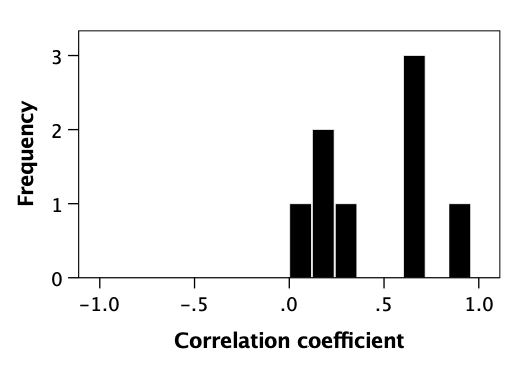
**

If there was no relationship between the two variables, the data would be normally distributed around τ=0. It clearly is not.

**References**

Arcand, B. (1973). *The Urgent Situation of the Cuiva Indians of Colombia.* International Work Group for Indigenous Affairs, Copenhagen (Denmark).

Brumbaugh, R. C. (1980). Models of Separation and a Mountain Ok Religion. *Ethos* 8: 332-348.

Beckerman, S., Erickson, P. I., Yost, J., Regalado, J., Jaramillo, L., Sparks, C., ... & Long, K. (2009). Life histories, blood revenge, and reproductive success among the Waorani of Ecuador. *Proceedings of the National Academy of Sciences*, *USA,* 106: 8134-8139.

Binford, L.R. (2019). *Constructing Frames of Reference*. University of California Press.

Bowles, S. (2009). Did warfare among ancestral hunter-gatherers affect the evolution of human social behaviors? *Science* 324: 1293-1298.

Bugos, P.E. (1985). *An Evolutionary Ecological Analysis of the Social Organization of the Ayoreo of the Northern Gran Chaco.* PhD thesis, Northwestern University.

Carneiro, R.L. (1967). On the relationship between size of population and complexity of social organization.  *Journal of Anthropological Research* 23: 234-243.

Carneiro, R.L. (1968). Ascertaining, testing, and interpreting sequences of cultural development. *Journal of Anthropological Research* 24: 254-274.

De Castro, E. (1992). *From the Enemy's Point of View*. Oxford: Oxford University Press.

Chagnon, N.A. (1968). Yanomamö: The Fierce People. New York: Holt Rinehart & Winston.

Conklin, B.A. (1995). “Thus are our bodies, thus was our custom”: mortuary cannibalism in an Amazonian society. *American Ethnologist* 22: 75-101.

Cook, E.A. (1967). Manga social organisation. PhD thesis, Yale University.

Diez, A.P., & Salzano, F.M. (1978). Evolutionary implications of the ethnography and demography of Ayoreo Indians. *Journal of Human Evolution* 7: 253-268.

Dunbar, R.I.M. (1993). Coevolution of neocortex size, group size and language in humans. *Behavioral and Brain Sciences* 16: 681-735.

Early, J.D., & Peters, J.F. (2000). *The Xilixana Yanomami of the Amazon: History, Social Structure, and Population Dynamics*. University Press of Florida.

Espinoza, U. (2020). *Intuitions on Ownership Among the Achuar of Southeastern Ecuador*. PhD thesis: UCLA.

Glasse, R.M. (1968). *Huli of Papua: A Cognatic Descent System*. The Hague: Mouton.

Gurven, M. & Kaplan, H. (2007). Longevity among hunter‐gatherers: a cross‐cultural examination. *Population and Development Review* 33: 321-365.

Gurven, M., Hill, K., Kaplan, H., Hurtado, A. & Lyles, R. (2000). Food transfers among Hiwi foragers of Venezuela: Tests of reciprocity. *Human Ecology* 28: 171-218.

Hallpike, C. R. (1986). Social and biological evolution II. Some basic principles of social evolution. *Journal of Social and Biological Structures* 9: 5-31.

Hart, C.W.M. (1930). The Tiwi of Melville and Bathurst Islands. *Oceania* 1: 167-180.

Headland, T.N. (1987). Kinship and social behavior among Agta Negrito hunter-gatherers. *Ethnology* 26: 261-280.

Headland, T. N. (1989). Population decline in a Philippine Negrito hunter‐gatherer society. *American Journal of Human Biology*, *1*(1), 59-72.

Heider, K. (1970). *The Dugum Dani*. Chicago: Aldine.

Heider, K.G. (2017). *The Dugum Dani: A Papuan Culture in the Highlands of West New Guinea*. Transaction Publishers.

Hiatt, L.R. (1965). *Kinship and Conflict: A Study of an Aboriginal Community in Northern Arnhem Land*. Canberra: Australian National University.

Hill, K. & Hurtado, A.M. (1996). *Ache Life History: The Ecology and Demography of a Foraging People*. New York: Aldine de Gruyter.

Hill, K., Hurtado, A.M. & Walker, R.S. (2007). High adult mortality among Hiwi hunter-gatherers: Implications for human evolution. *Journal of Human Evolution* *52*: 443-454.

Huber, P.B. (1973). Defending the cosmos: violence and social order among the Anggor of New Guinea. In: M. Nettieship, R. Givens & A. Nettieship (eds.) *War, Its Causes and Correlates*, pp. 619-661. The Hague: Mouton.

Huber, P.B. (1980). The Anggor bowman: ritual and society in Melanesia. *American Ethnologist* 7: 43-57.

Johnson, A. & Behrens, C.A. (1982). Nutritional criteria in Machiguenga food production decisions: a linear-programming analysis. *Human Ecology* 10: 167-189.

Keeley, L. H. (1996). *War Before Civilization: The Myth of the Peaceful Savage*. Oxford: Oxford University Press

Knauft, B.M. (1987). Reconsidering violence in simple human societies: homicide among the Gebusi of New Guinea. *Current Anthropology* 28: 457-500.

Lee, R.B. (1972). !Kung spatial organization: an ecological and historical perspective. *Human Ecology* 1: 125-147.

Lee, R.B. (1979). *The !Kung San: Men, Women and Work in a Foraging Society*. Cambridge: Cambridge University Press.

Lindgärde, F., Widén, I., Gebb, M., & Ahrén, B. (2004). Traditional versus agricultural lifestyle among Shuar women of the Ecuadorian Amazon: effects on leptin levels. *Metabolism* 53: 1355-1358.

Macfarlan, S.J., Walker, R.S., Flinn, M.V. & Chagnon, N. A. (2014). Lethal coalitionary aggression and long-term alliance formation among Yanomamö men. *Proceedings of the National Academy of Sciences*, USA, 111: 16662-16669.

Macfarlan, S.J., Erickson, P.I., Yost, J., Regalado, J., Jaramillo, L. & Beckerman, S. (2018). Bands of brothers and in-laws: Waorani warfare, marriage and alliance formation. *Proceedings of the Royal Society, London,* 285B: 20181859.

Mader, E., & Gippelhauser, R. (2000). Power and kinship in Shuar and Achuar society. In: J.F. Collier (ed.) *Dividends of Kinship. Meanings and Uses of Social Relatedness*, pp. 61-91. London: Routledge.

Mandelbaum, D.G. (1979). *The Plains Cree: An Ethnographic, Historical and Comparative Study*. Regina: University of Regina.

Marlowe, F. (2002). Why the Hadza are still hunter-gatherers. In; S. Kent (ed.) *Ethnicity, Huntergatherers, and the ‘Other’*, pp. 247-81. Washington DC: Smithsonian Institution.

Marlowe, F. (2010). *The Hadza: Hunter-Gatherers of Tanzania*. Oakland CA: University of California Press.

Meggitt, M. (1957). Enga political organisatuion: a preliminary description. *Mankind* 5: 133-137.

Meehan, B.F. (1975). *Shell Bed to Shell Midden.* PhD thesis, Australian National University.

Minter, T. (2017). Mobility and sedentarization among the Philippine Agta. *Senri Ethnological Studies* 95 119-150.

Nivette, A.E. (2011). Violence in non-state societies: A review. *The British Journal of Criminology* 51: 578-598.

Orr, C.M., Dufour, D.L. & Patton, J.Q. (2001). A comparison of anthropometric indices of nutritional status in Tukanoan and Achuar Amerindians. *American Journal of Human Biology* 13: 301-309.

Picchi, D. (1995). Village division in lowland South America: the case of the Bakairí Indians of central Brazil. *Human Ecology* 23: 477-498.

Rival, L. (1993). The growth of family trees: understanding Huaorani perceptions of the forest. *Man* 28: 635-652.

Robarchek, C.A. & Robarchek, C.J. (1992). A comparative study of Waorani and Semai. J. Silverberg & J.P. Gray (eds.) *Aggression and Peace in Humans and Other Primates*, pp. 188-213. New York: Oxford University Press.

Roser, M. (2013). *Ethnographic and Archaeological Evidence on Violent Deaths.*  <https://ourworldindata.org/ethnographic-and-archaeological-evidence-on-violent-deaths?mod=article_inline>

von Rueden, C. (2011). *The Acquisition of Social Status by Males in Small-Scale Human Societies*. PhD thesis, University of California Santa Barbara.

von Rueden, C., Gurven, M., Kaplan, H. & Stieglitz, J. (2014). Leadership in an egalitarian society. *Human Nature* 25: 538-566.

Schiefenhovel, W. (1998). The Eipo of the Highlands. In: I. Eibl-Eibesfeldt & F.K. Salter (eds.) *Ethnic Conflict and Indoctrination: Altruism and Identity in Evolutionary Perspective*, pp. 109-132. Oxford: Berghahn.

Shankman, P. (1991). Culture contact, cultural ecology, and Dani warfare. *Man* 29:299-321.

Sobel, E., & Bettles, G. (2000). Winter hunger, winter myths: subsistence risk and mythology among the Klamath and Modoc. *Journal of Anthropological Archaeology*,19: 276-316.

Somerville, A.D., Martin, M.A., Hayes, L P., Hayward, D., Walker, P.L., Schoeninger, M.J., ... & Stojanowski, C.M. (2017). Exploring patterns and pathways of dietary change: Preferred foods, oral health, and stable isotope analysis of hair from the Dani of Mulia, Papua, Indonesia. *Current Anthropology* 58: 31-56.

Steadman, L. B. (1971). Neighbours and killers: residence and dominance among the Hewa of New Guinea. PhD thesis: Australian National University.

Tung, T. A. (2007). Trauma and violence in the Wari empire of the Peruvian Andes: warfare, raids, and ritual fights. *American Journal of Physical Anthropology: The Official Publication of the American Association of Physical Anthropologists*, *133*(3).

Turner, T. (2003). The beautiful and the common: inequalities of value and revolving hierarchy among the Kayapó. *Tipití* 1: 11-26.

Walker, R.S. & Bailey, D.H. (2013). Body counts in lowland South American violence. *Evolution and Human Behavior* 34: 29–34.

Walker, R.S. & Hill, K.R. (2014). Causes, consequences, and kin bias of human group fissions. *Human Nature* *25*: 465-475.

Werner, D. W. (1980). *The Making of a Mekranoti Chief: The Psychological and Social Determinants of Leadership in a Native South American Society*. PhD thesis: City University of New York.
